# The Concavity of the Maximal Expiratory Flow–Volume Curve Reflects the Extent of Emphysema in Obstructive Lung Diseases

**DOI:** 10.1038/s41598-019-49591-2

**Published:** 2019-09-11

**Authors:** Fumi Mochizuki, Hiroaki Iijima, Azusa Watanabe, Naoya Tanabe, Susumu Sato, Masanari Shiigai, Keiji Fujiwara, Takafumi Shimada, Hiroichi Ishikawa, Jun Kanazawa, Yohei Yatagai, Hironori Masuko, Tohru Sakamoto, Shigeo Muro, Nobuyuki Hizawa

**Affiliations:** 1 Department of Respiratory Medicine, Tsukuba Medical Centre Hospital, Tsukuba, Japan; 2 Department of Radiology, Tsukuba Medical Centre Hospital, Tsukuba, Japan; 3 Department of Respiratory Medicine, Graduate School of Medicine, Kyoto University, Kyoto, Japan; 4 Department of Pulmonary Medicine, Faculty of Medicine, University of Tsukuba, Tsukuba, Japan; 5 Department of Respiratory Medicine, Nara Medical University, Kashihara, Japan

**Keywords:** Asthma, Chronic obstructive pulmonary disease

## Abstract

A concave-shaped maximal expiratory flow-volume (MEFV) curve is a spirometric feature in chronic obstructive pulmonary disease (COPD). The MEFV curve is characterized by an increase in the Obstructive Index, which is defined as a ratio of forced vital capacity to the volume-difference between two points of half of the peak expiratory flow on the MEFV curve. We hypothesized that the Obstructive Index would reflect the severity of emphysema in patients with COPD and asthma-COPD overlap (ACO). Thus, the aim of this retrospective study was to evaluate whether the Obstructive Index on spirometry is associated with the extent of emphysema on computed tomography (CT) in patients with COPD, ACO, and asthma (N = 65, 15, and 53, respectively). The percentage of low-attenuation volume (LAV%) and wall area (WA%) were measured on CT. The Obstructive Index was higher in patients with COPD and ACO than in those with asthma. Spearman correlation showed that a greater Obstructive Index was associated with a higher LAV%, but not WA%. Multivariate analysis showed that Obstructive Index was associated with LAV% (standardized β = 0.43, *P* < 0.0001) independent of other spirometric indices. The Obstructive Index is a useful spirometric index that reflects the extent of emphysema.

## Introduction

Chronic obstructive pulmonary disease (COPD) is a major concern worldwide, as its prevalence and mortality rate continue to increase, imposing a huge economic burden^1,2^. The heterogeneity of COPD further complicates its clinical management^3^. It can be classified into emphysema or non-emphysema phenotypes, which are easily identifiable on computed tomography (CT). Numerous CT studies have shown that the emphysema phenotype is associated with a rapid decline in forced expiratory volume in 1 second (FEV_1_)^4^, frequency of exacerbations^5^, a poor prognosis^6^, osteoporosis^7^, occurrence of lung cancer^8^, loss of skeletal muscle mass^9^, and reduction in body mass index (BMI)^10^. These findings emphasize the importance of identifying the emphysema phenotype in the early stage of the disease; however, chest CT scans entail radiation exposure^11^ and are not always available in primary care facilities. In addition, although it is possible to estimate the extent of emphysema by measuring diffusion capacity, this physiological examination also requires large equipment and it is impractical to routinely perform this examination for all patients suspected of having obstructive lung diseases.

Spirometry is widely used for low-cost management of COPD and non-invasively measures lung function parameters, such as FEV_1_ and forced vital capacity (FVC). In addition, it allows for the visualization of the maximal expiratory flow-volume (MEFV) curve. MEFV curves with varying concave contours are seen in patients with COPD, and previous reports have shown that this concave shape was partly attributable to loss of elastic recoil^12,13^.

Therefore, we hypothesized that it would be possible to predict the extent of emphysema among patients with obstructive lung diseases by using a simple spirometry measurement to obtain the Obstructive Index, initially proposed in Japan in 1978, which expresses the concavity of MEFV curves even in patients who have low FVC^14^. In this study, we explored the usefulness of the Obstructive Index by comparing quantitative CT (QCT) of lung emphysema with visual assessment of MEFV curves in patients with chronic obstructive lung diseases.

## Results

**Figure 1:**
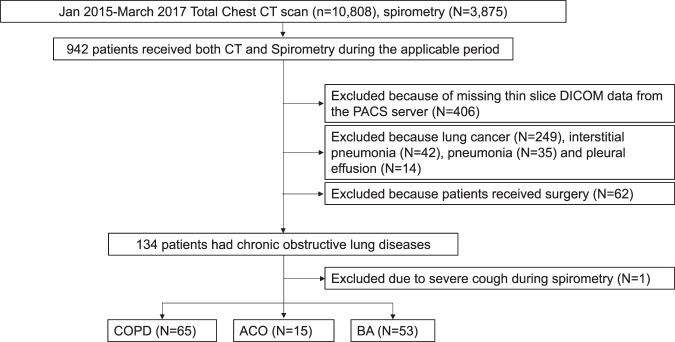
Chart flow of patient’s selection. ACO, asthma–COPD overlap; BA, bronchial asthma; COPD, chronic obstructive pulmonary disease; CT, computed tomography; DICOM, digital imaging and communications in medicine; PACS, picture archiving and communication systems.

Between January 1, 2015, and March 31, 2017, 10,808 patients had a chest CT scan and 3,875 patients had a spirometry test at the Tsukuba Medical Centre Hospital. Among them, 942 patients had both a chest CT scan and a spirometry test within a 3-month period on the same day or on separate occasions. Four hundred and six patients were excluded because their thin slice digital imaging and communications in medicine (DICOM) data were not available. Furthermore, patients with lung cancer (N = 249), interstitial pneumonia (N = 42), bacterial pneumonia (N = 35) and pleural effusion (N = 14), as well as 62 patients who had surgery were excluded. Finally, one patient with a severe cough during spirometry was excluded. A total of 133 patients who had COPD, asthma–COPD overlap (ACO), or bronchial asthma (BA) were subsequently analyzed for the current study (Fig. 1)

### Baseline characteristics

Table 1 shows the characteristics of subjects and the results of univariate analyses of the nonparametric test, including the Obstructive Index in each group. In the Steel-Dwass test, the Obstructive Index in the BA group was lower than those in the COPD and ACO groups (*P* < 0.0001 and *P* = 0.0001). The half of the peak expiratory flow (PEF), i.e., the reference point of the Obstructive Index, was larger than the forced expiratory flow at 50% (FEF_50_), which reflects the concavity of the MEFV curve in COPD or ACO compared to BA, suggesting that half of the PEF is reached faster than the halfway volume of the MEFV curve, FEF_50,_ in COPD or ACO. In terms of QCT measurements, the percentage of low attenuation volume (LAV%) was highest in the COPD group, followed by those in the ACO and BA groups, while there was no difference in the percentage of wall area (WA%) of the right apical segmental bronchus (B^1^) or right anterior basal segmental bronchus (B^8^) among the disease groups.

**Table 1:** Characteristics of subjects.

Characteristic	ALL (n = 133)	COPD (n = 65)	ACO (n = 15)	BA (n = 53)	*P* (Kruskal-Wallis test)	*P* (COPD vs ACO)	*P* (ACO vs BA)	*P* (COPD vs BA)
Age, y	70 (61 to 75)	73 (67 to 79)	74 (64 to 78)	61 (50 to 71)	<0.0001	0.943	0.008	<0.0001
Male, N (%)	99 (74.4)	59 (90.8)	13 (86.7)	27 (50.9)	<0.0001			
Height, cm	163.0 (156.0 to 169.0)	164.0 (158.0 to 169.5)	165.0 (156.1 to 168.0)	160.0 (152.5 to 169.8)	0.492	0.976	0.907	0.449
BMI, kg/m^2^	22.4 (20.6 to 25.0)	22.1 (19.5 to 23.4)	22.7 (20.2 to 26.2)	24.2 (21.5 to 26.5)	0.009	0.529	0.716	0.007
Current smoker, No. (%)	31 (23.3)	20 (30.8)	2 (13.3)	9 (17.0)	<0.0001			
SI, Pack-year	27.5 (0 to 51.3)	44.3 (26.8 to 66.0)	45.0 (13.0 to 80.0)	0 (0 to 22.0)	<0.0001	0.847	0.002	<0.0001
FVC, L	2.73 (2.07 to 3.37)	2.63 (2.12 to 3.29)	2.73 (1.60 to 3.41)	2.80 (2.07 to 3.54)	0.348	0.590	0.350	0.721
FVC% predicted	83.0 (66.7 to 95.5)	78.5 (65.5 to 94.5)	73.3 (49.7 to 93.1)	87.2 (76.7 to 100.8)	0.012	0.239	0.035	0.078
FEV_1_, L	1.64 (1.16 to 2.20)	1.35 (1.06 to 1.92)	1.29 (0.80 to 1.73)	2.11 (1.55 to 2.80)	<0.0001	0.828	0.001	<0.0001
FEV_1_% predicted	66.2 (48.1 to 85.9)	55.0 (40.8 to 71.4)	49.2 (32.2 to 64.0)	82.9 (69.3 to 98.0)	<0.0001	0.717	<0.0001	<0.0001
FEV_1_/FVC ratio	62.8 (52.0 to 73.1)	57.0 (40.9 to 63.3)	52.6 (48.3 to 60.7)	75.4 (70.4 to 80.2)	<0.0001	0.905	<0.0001	<0.0001
Obstructive Index	3.54 (2.44 to 5.68)	4.82 (3.41 to 7.69)	4.42 (3.48 to 6.13)	2.34 (1.90 to 3.10)	<0.0001	0.801	0.0001	<0.0001
FEF_25–75_, L/s	0.83 (0.49 to 1.65)	0.57 (0.35 to 0.96)	0.63 (0.31 to 0.78)	1.71 (1.19 to 2.49)	<0.0001	0.905	<0.0001	<0.0001
FEF_25–75_/FVC	0.32 (0.20 to 0.51)	0.22 (0.15 to 0.32)	0.23 (0.18 to 0.28)	0.61 (0.44 to 0.80)	<0.0001	0.986	<0.0001	<0.0001
Δ(0.5PEF-FEF_50_), L/S	0.85 (0.23 to 1.52)	1.01 (0.57 to 1.72)	1.16 (0.23 to 1.79)	0.29 (−0.59 to 1.14)	<0.0001	0.985	0.020	<0.0001
LAV, ml	711.6 (91.9 to 1530.1)	1287.1 (565.7 to 2157.9)	534.2 (133.7 to 1504.1)	95.3 (13.5 to 862.0)	<0.0001	0.119	0.078	<0.0001
LAV%	15.3 (2.15 to 29.9)	23.5 (12.9 to 40.3)	11.6 (2.1 to 26.2)	2.7 (0.4 to 15.9)	<0.0001	0.113	0.068	<0.0001
Right B^1^ WA%	43.0 (34.0 to 52.0)	43.0 (34.0 to 52.0)	47.0 (35.0 to 53.0)	44.0 (34.5 to 52.5)	0.763	0.758	0.792	0.994
Right B^8^ WA%	43.0 (33.0 to 51.0)	44.0 (34.0 to 52.0)	42.0 (31.0 to 50.0)	42.0 (32.0 to 51.5)	0.616	0.687	0.946	0.737

Data are presented as medians and interquartile ranges. The predicted values of FVC and FEV_1_ were calculated with the LMS methods^30^. *P* values less than 0.05 were considered significant.

ACO, asthma–COPD overlap; BA, bronchial asthma; BMI, body mass index; COPD, chronic obstructive pulmonary disease; FEF_25–75_, forced expiratory flow between 25 and 75%; FEF_50_, forced expiratory flow at 50%; FEV_1_, forced expiratory volume in 1 second; FVC, forced vital capacity; LAV, low attenuation volume (<−960 Hounsfield Units); LAV%, the percentage of LAV to total lung volume measured by QCT; LMS, lambda-mu-sigma; L, liter; L/s, liter/second; PEF, peak expiratory flow; QCT, quantitative computed tomography; Right B^1^, right apical segmental bronchus; Right B^8^, right anterior basal segmental bronchus; SI, smoking index; WA%, the percentage of wall area.

### Relationship of MEFV curve concavity on emphysematous change

**Figure 2:**
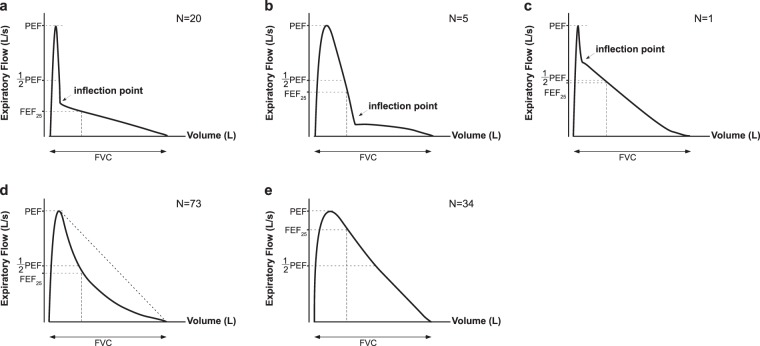
Schematic representation of MEFV curves. The AC (**a**) has an abrupt decrease in flow rate and an inflection point that occurs at less than 50% of the PEF and within the first 25% of the FVC^12^. The Int (**b**,**c**) is like the AC but meets only 1 of the AC criteria^13^. The C (**d**) exhibits a gradual decrease in the descending limb of the MEFV curve. N (**e**) is the normal MEFV curve. The visual patterns of the MEFV curves were established by a consensus reading by 3 respiratory physicians (FM, KF, and TS). AC, airway collapse; C, curvilinear; FEF_25_, forced expiratory flow at 25%, FVC, forced vital capacity; Int, intermediate; MEFV, maximal expiratory flow-volume; N, normal, PEF, peak expiratory flow.

The classification of MEFV curves is shown in Fig. 2. We found that patients with the airway collapse (AC) pattern had the most severe emphysema, followed by those with the intermediate (Int), curvilinear (C), and normal (N) patterns (Table 2). There was no difference in the WA% between the types of MEFV curves. Each type of MEFV curve was distinguished by the Obstructive Index values. We also examined the presence or absence of the inflection point in the descending limb of the MEFV curves. Nominal logistic regression analysis showed that LAV% was the significant factor associated with the presence of an inflection point on MEFV curves (Table 3).

**Table 2:** Relationship between visual assessment of the MEFV curve, the Obstructive Index, emphysema, and airway wall thickness.

	AC (n = 20)	Int (n = 6)	C (n = 73)	N (n = 34)	*P* (All Groups Omnibus Test)	*P* (AC vs Int)	*P* (AC vs C)	*P* (C vs N)
Obstructive Index	7.92 (6.18 to 10.90)	4.91 (3.92 to 6.33)	3.81 (2.91 to 4.95)	2.23 (1.70 to 2.49)	<0.0001	0.034	<0.0001	<0.0001
LAV%	37.3 (22.8 to 53.5)	21.0 (17.4 to 40.1)	15.6 (3.9 to 25.0)	2.1 (0.58 to 13.0)	<0.0001	0.764	0.002	0.002
Right B^1^ WA%	45.5 (32.0 to 52.0)	39.5 (30.0 to 59.8)	42.0 (35.0 to 51.0)	47.0 (37.3 to 55.0)	0.576	0.987	0.972	0.496
Right B^8^ WA%	42.5 (32.0 to 50.3)	39.0 (20.5 to 60.8)	43.0 (33.0 to 51.5)	42.5 (32.8 to 52.0)	0.953	0.987	0.988	1.000

Data are presented as medians and interquartile ranges. All MEFV curves were assessed by a consensus reading of 3 respiratory physicians. The AC has an abrupt decrease in flow rate and an inflection point at less than 50% of peak flow rate and within the first 25% of FVC^12^. The Int was similar to the AC but met only 1 of the AC criteria^13^. The C exhibited a gradual decrease in the descending limb of the MEFV curve^13^. The schematic representation of the type of MEFV curves is shown in Fig. 2. *P* values less than 0.05 were considered significant.

AC, airway collapse; C, curvilinear; Int, intermediate; MEFV, maximal expiratory flow-volume; N, normal.

**Table 3:** Nominal logistic regression analysis results of MEFV curves which exhibit inflection point using clinical characteristics and QCT measurements.

	LR χ^2^	*P* value
Age (y)	0.69	0.4049
Female	0.55	0.4570
Height (cm)	2.50	0.1139
BMI (kg/m^2^)	0.91	0.3397
SI, ≥10 pack-years	0.98	0.3234
Current smoker	1.95	0.1631
LAV%	13.25	0.0003
Right B^1^ WA (%)	0.06	0.8087
Right B^8^ WA (%)	0.19	0.6641

The target level of the dependent variable is the presence of inflection point in the descending limb of MEFV curve (AC and Int *vs* C and N). *P* values less than 0.05 were considered significant.

AC, airway collapse; BMI, body mass index; C, curvilinear; Int, intermediate; LR χ^2^, likelihood ratio Chi-square; MEFV, maximal expiratory flow-volume; N, normal; QCT, quantitative computed tomography; SI, smoking index.

### Bivariate correlation analysis

The Obstructive Index was significantly correlated with the LAV% (*ρ* = 0.56, *P* < 0.0001), but not with the WA%. FEV_1_ (*ρ* = −0.34, *P* < 0.0001), FEV_1_% predicted (*ρ* = −0.36, *P* < 0.0001), forced expiratory flow between 25 and 75% (FEF_25–75_), (*ρ* = −0.47, *P* < 0.0001), and FEF_25–75_/FVC (*ρ* = −0.56, *P* < 0.0001) were also related to the LAV%. FEV_1_ and FEV_1_% of the predicted were weakly correlated with the right B^1^ WA% (*ρ* = −0.32, *P* = 0.0002, and *ρ* = −0.22, *P* = 0.012, respectively) (Table 4).

**Table 4:** Spearman’s rank correlation coefficients between spirometric and QCT measurements.

	LAV%		Right B^1^ WA%		Right B^8^ WA%	
	*ρ*	*P* value	*ρ*	*P* value	*ρ*	*P* value
FEV_1_, L	−0.34	<0.0001	−0.32	0.0002	−0.07	0.436
FEV_1_% predicted	−0.36	<0.0001	−0.22	0.012	−0.07	0.415
Obstructive Index	0.56	<0.0001	−0.02	0.844	−0.04	0.670
FEF_25–75_, L/s	−0.47	<0.0001	−0.19	0.031	−0.05	0.576
FEF_25–75_/FVC	−0.56	<0.0001	−0.04	0.616	−0.03	0.726

*P* values less than 0.05 were considered significant.

FEF_25–75_, forced expiratory flow between 25 and 75%; FEV_1_, forced expiratory volume in 1 second; FVC, forced vital capacity; L/s, liter/second; Right B^1^, right apical segmental bronchus; Right B^8^, right anterior basal segmental bronchus.

### The relationship between Obstructive Index, FEV_1_% predicted and FEF_25–75_/FVC (Fig. 3)

**Figure 3:**
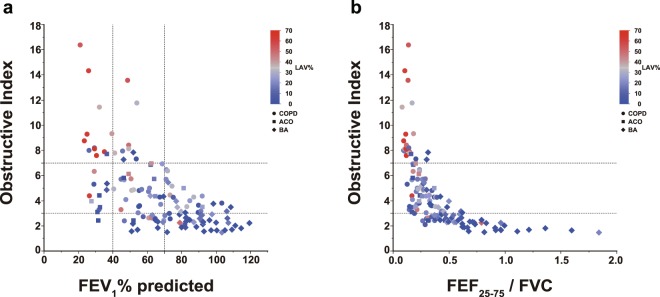
Correlation of the Obstructive Index with FEV_1_% predicted and FEF_25–75_/FVC. (**a**) Although a linear correlation was found between the Obstructive Index and FEV_1_% predicted in patients who had high FEV_1_% predicted, the linearity disappeared and the Obstructive Index was high in COPD patients who had low FEV_1_% predicted. (**b**) The relationship between the Obstructive Index and FEF_25–75_/FVC^32^ was hyperbolic. COPD, chronic obstructive pulmonary disease; FEV_1_%, forced expiratory volume in 1 second; FEF_25–75_, forced expiratory flow between 25 and 75%; FVC, forced vital capacity.

A linear correlation was found between the Obstructive Index and FEV_1_% predicted overall; the Obstructive Index values were particularly high in COPD patients who had lower FEV_1_% predicted and higher LAV% (Fig. 3a). The relationship between the Obstructive Index and FEF_25–75_/FVC was hyperbolic (Fig. 3b). The Obstructive Index values were again particularly high in COPD patients with lower FEF_25–75_/FVC and higher LAV% values.

### Factors associated with emphysema on multivariate regression analysis

The most striking association was found between LAV% and the Obstructive Index (standardized β = 0.43, *P* < 0.0001), which was independent of FEV_1_% predicted, FEF_25–75_/FVC, and lower FEV_1_/FVC, as judged by the lower limit of normal for the FEV_1_/FVC ratio. BMI was significantly inversely associated with the extent of emphysema (standardized β = −0.28, *P* < 0.0001). These correlations of LAV% with the Obstructive Index and BMI remained significant for a subgroup consisting of patients with both COPD and with ACO (standardized β = 0.32; *P* = 0.0025, β = −0.32; *P* < 0.0001, respectively), and for a subgroup consisting of only patients with COPD only (standardized β = 0.33; *P* = 0.0059, β = −0.27; *P* = 0.0019, respectively) **(**Table 5).

**Table 5:** Multivariate regression analysis with LAV% as the dependent variable.

	All		COPD + ACO		COPD	
	*R*^2^ = 0.622, *P* < 0.0001		*R*^2^ = 0.674, *P* < 0.0001		*R*^2^ = 0.700, *P* < 0.0001	
	standardized β	*P* value	standardized β	*P* value	standardized β	*P* value
Age, y	0.01	0.913	0.05	0.644	0.10	0.363
Female	−0.20	0.0461	−0.06	0.592	−0.03	0.756
Height, cm	−0.16	0.105	−0.02	0.867	0.02	0.832
BMI, kg/m^2^	−0.28	<0.0001	−0.32	<0.0001	−0.27	0.0019
SI, >10 pack-years	0.07	0.358	−0.09	0.267	−0.17	0.046
Current smoker	−0.05	0.438	−0.03	0.706	0.00	0.985
FEV_1_% predicted	−0.08	0.388	−0.17	0.098	−0.23	0.070
Obstructive Index	0.43	<0.0001	0.32	0.0025	0.33	0.0059
FEF_25–75_/FVC	0.07	0.483	−0.11	0.413	−0.07	0.654
FEV_1_/FVC <LLN	0.04	0.619	0.07	0.420	0.08	0.398
CT scanner, Aquilion	0.38	<0.0001	0.47	<0.0001	0.42	<0.0001

The LLN of FEV_1_/FVC were calculated with the LMS method^30^. *P* values less than 0.05 were considered significant.

ACO, asthma–COPD overlap; BMI, body mass index; COPD, chronic obstructive pulmonary disease; FEF_25–75_, forced expiratory flow between 25 and 75%; FEV_1_, forced expiratory volume in 1 second; FVC, forced vital capacity; LLN, lower limit of normal; SI, smoking index.

### Comparison of QCT measurements by degree of FEV_1_% predicted

**Figure 4:**
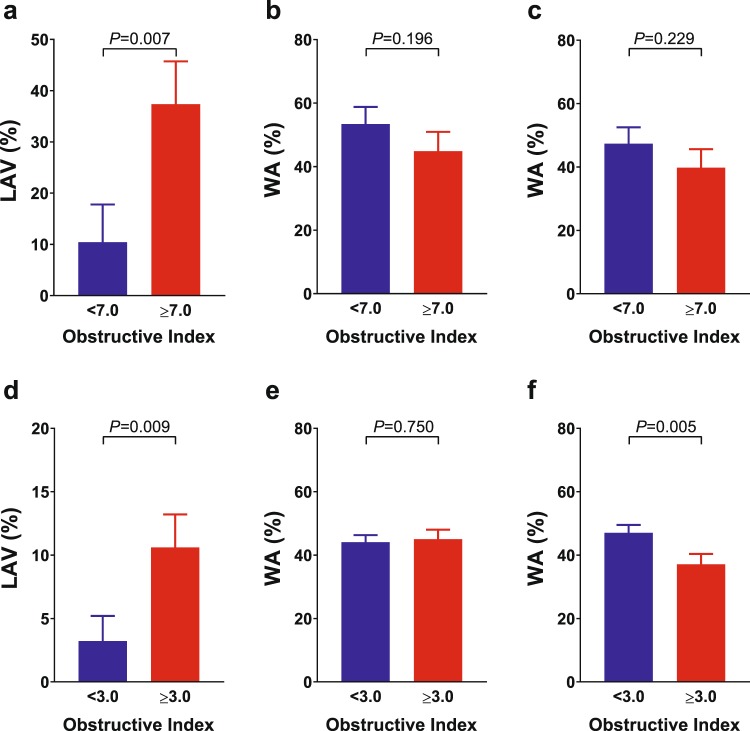
Comparison of QCT measurements at the cut-off value of Obstructive Index by the degree of airway obstruction. Bars represent LS mean ± SEM. Values are adjusted by age, sex, height, smoking index, smoking status, and CT scanner type. There was a difference between LAV% and the Obstructive Index in patients who had either a low FEV_1_% predicted (≤40%, **a**–**c**) or a high FEV_1_% predicted (≥70%, **d**–**f**). The Obstructive Index <7.0, N = 11 (ACO = 5, BA = 2, COPD = 4); the Obstructive Index ≥7.0, N = 12 (ACO = 1, BA = 0, COPD = 11); the Obstructive Index <3.0, N = 36 (ACO = 0, BA = 31, COPD = 5), the Obstructive Index ≥3.0, N = 24 (ACO = 2, BA = 8, COPD = 14) ACO, asthma–COPD overlap; BA, bronchial asthma; COPD, chronic obstructive pulmonary disease; CT, computed tomography; FEV_1_, forced expiratory volume in 1 second; LAV, low attenuation volume; LS, least squares; QCT, quantitative CT; SEM, standard error of the mean; WA%, the percentage of wall area.

We also compared the LAV% or WA% of the right B^1^ or right B^8^ according to the degree of FEV_1_% predicted and cut-off level of Obstructive Index (Fig. 4a–f). There was a significant difference in LAV% at the cut-off level of 7.0 Obstructive Index in patients who had an FEV_1_% predicted ≤40% (*P* = 0.007, Fig. 4a), and either at a cut-off level of 3.0 Obstructive Index in patients with a high FEV_1_% predicted ≥70% (*P* = 0.009, Fig. 4d). Regarding WA%, a significant difference was observed in the right B^8^ at a cut-off level of 3.0 Obstructive Index in patients with a high FEV_1_% predicted ≥70% (*P* = 0.005, Fig. 4f).

### Optimal Obstructive Index cut-off values for predicting the presence of emphysema

**Figure 5:**
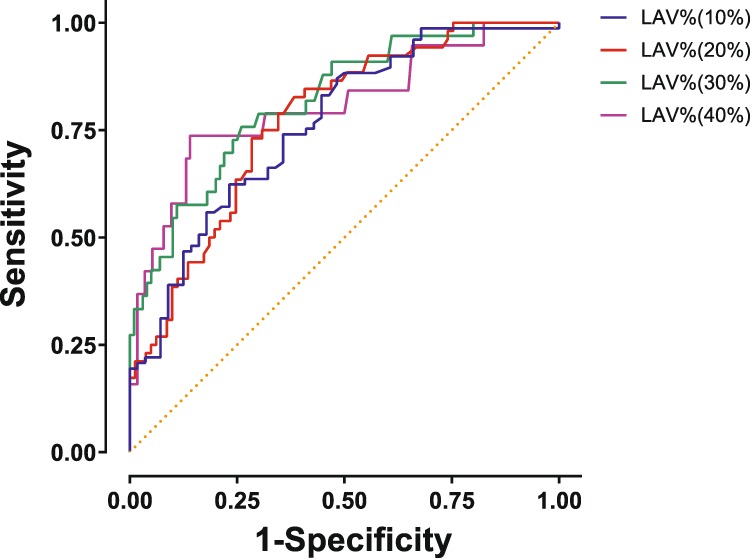
Comparison of LAV of total lungs for predicting the presence of emphysema by ROC analysis. We evaluated emphysema based on four stages of LAV%:10%, 20%, 30%, and 40%. ROC analysis showed that a LAV of 30% and an Obstructive Index of 4.38 were the optimal cut-off values for determining the presence of emphysema in patients with obstructive lung diseases (Table 6). LAV, low attenuation volume; ROC, receiver operating characteristic.

To determine the optimal Obstructive Index cut-off values for predicting the presence of emphysema, the area under the curves (AUCs) for receiver operating characteristic (ROC) curves by levels of LAV% were calculated (Fig. 5 and Table 6). We evaluated four stages of LAV%: 10%, 20%, 30%, and 40%. The AUC at a LAV% of 30% was 0.819 (95% CI; 0.736–0.901) and the corresponding cut-off value of the Obstructive Index for predicting emphysema was 4.38 (Table 6).

**Table 6:** Obstructive Index-based prediction of emphysema progression by area under the receiver-operating characteristic curve analysis.

Definition of emphysema	AUC	95% CI	Youden Index	Obstructive Index threshold
LAV 10%	0.762	0.681 to 0.843	0.391	3.89
LAV 20%	0.773	0.695 to 0.852	0.447	3.89
LAV 30%	0.819	0.736 to 0.901	0.498	4.38
LAV 40%	0.806	0.686 to 0.926	0.596	5.74

AUC, area under the curve; CI, confidence interval; LAV, the percentage of low attenuation volume to total lung volume measured by quantitative computed tomography.

## Discussion

This study investigated whether the Obstructive Index, measured spirometrically, could be used to predict the extent of emphysema, as measured on CT, using cross-sectional data of patients with COPD, ACO, or BA; this has not been reported previously. Previous physiological studies showed that concave MEFV curves on spirometry are common in patients with COPD and that this shape reflects the loss of elastic recoil; however, no report to date has investigated its association with the severity of emphysema. Therefore, the present study substantially extended this body of knowledge by revealing that Obstructive Index is closely correlated with the extent of emphysematous change on CT, independently of FEV_1_% predicted, FEF_25–75_/FVC, and a lower FEV_1_/FVC ratio. This association was preserved even when the same analyses were performed in a subgroup including patients with COPD only or a subgroup including patients with both COPD and with ACO or with BA (Supplementary Tables S1–S5 and Figs S1–3). Furthermore, the Obstructive Index closely linked visual assessment of concavity of the MEFV curve likewise the extent of emphysema.

The concavity in the descending limb of the MEFV curve is a recognized feature of obstructive lung diseases^14–19^. The earliest change in small airways are thought to be reflected in the terminal portion of the MEFV curve^20^ and quantitatively, the FEF_25–75_ was reported to reflect concavity and small airway narrowing^20–22^.

The relationship between FEF_25–75_/FVC and Obstructive Index is shown in Fig. 3b; each parameter could describe different obstructive impairment features. In bivariate analysis, FEF_25–75_, FEF_25–75_/FVC, and Obstructive Index were associated with emphysema, while in multivariate analysis, the Obstructive Index was the only significant factor related to the extent of emphysema among spirometric indices. This suggests that the Obstructive Index would be a more appropriate measurement than FEF_25–75_ or FEF_25–75_/FVC for evaluating the extent of emphysema.

As COPD progresses, timed segments of spirometry, i.e., FEV_1_, decreases; FEV_1_ is used as an index of the severity of airway obstruction. We also examined the relationship between the Obstructive Index and the extent of emphysema by the degree of FEV_1_% predicted (Fig. 4) and found a persistent association between emphysema and the Obstructive Index, irrespective of the level of FEV_1_% predicted. Therefore, it is possible to differentiate patients with the phenotypic expression of emphysema from those with obstructive lung diseases, who have similar degrees of airway obstruction, by using the Obstructive Index.

**Figure 6:**
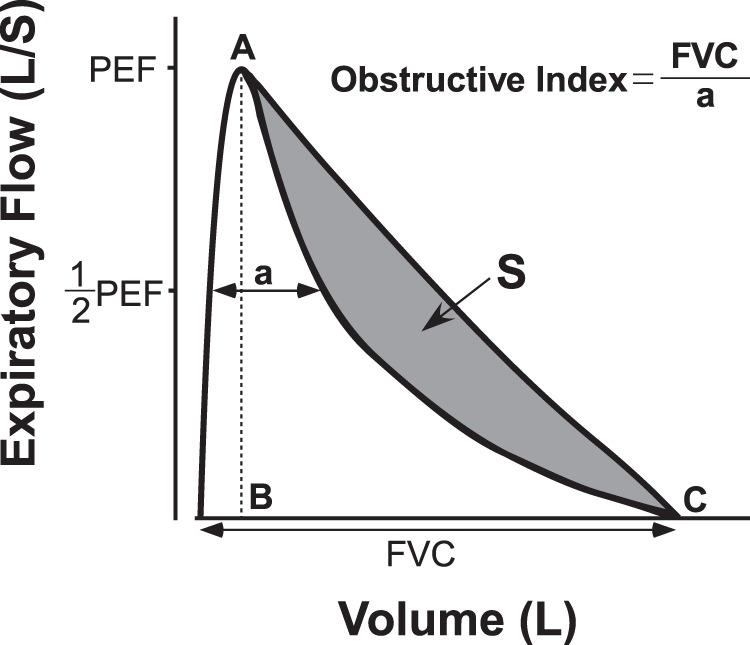
Schematic illustration of Obstructive Index. The Obstructive Index^14^ is defined as the numerator (FVC) divided by the denominator (“a”), which is the volume between the points at half-PEF on the MEFV curve. The Obstructive Index is reported to be correlated with the area ratio of S to triangle ABC even in patients with both obstructive and restrictive impairments, while the FEV_1_/FVC ratio is not correlated with the area ratio to triangle ABC in patients with restrictive impairments^14^. The S is defined as the area under the descending limb of the MEFV curve. The triangle ABC is formed by the top of the PEF (“A”) and the line perpendicular to X-axis “B” and the point of residual volume, “C.” This illustration is a modified reprint from *Jpn*. *J*. *Chest Dis*. 37, Ohsugi, T. *et al*., 956, A new index of the maximal expiratory flow-volume curve, 1978, with permission from KOKUSEIDO PUBLISHING. Copyright © KOKUSEIDO PUBLISHING. FEV_1_, forced expiratory volume in 1 second; FVC, forced vital capacity; MEFV, maximal expiratory flow-volume; PEF, peak expiratory flow.

We also explored the relationship between visual assessment of the MEFV curve and the extent of emphysema. The presence of an inflection point in the descending part of MEFV curves was associated with the extent of emphysema. Mechanistically, when airflow obstruction becomes more severe, the expiratory flow decreases more abruptly, to the level of half of the PEF, due to loss of elastic recoil, reduced central airway support, and increased peripheral resistance and pleural pressures during forced expiration^12,13^. In fact, although FEF_50_ also reflects the concavity of the MEFV curve^16^, we found that the half-PEF values were larger than the FEF_50_ values in patients with COPD or ACO (Table 1), suggesting that focusing on the MEFV curve at the half-PEF level is a rational approach for detecting earlier collapse of the MEFV curve. In addition, as the Obstructive Index is a ratio of FVC relative to “a” (Fig. 6), it could express the concavity more appropriately, even in patients who have decreased FVC, such as those with severe airflow obstruction.

This study also has some limitations. Firstly, although the expiratory effort could affect the PEF and FVC, which are components of the Obstructive Index, we did not assess the impact of the FVC maneuver on Obstructive Index computation. Jayamanne *et al*.^12^ assessed the effects of the graded effort vital capacity maneuver on patients with an AC pattern. They reported that with regard to the submaximal effort, defined by 50 to 60% of the maximal pleural pressure, there was no difference in the flow-volume curve when compared with the MEFV curve, whereas a minimal effort <40% of the pleural pressure resulted in the disappearance of the inflection point in the descending limb of the MEFV curve. In this study, as the spirometry was performed by well-trained technicians following the statement of American Thoracic Society (ATS) and the European Respiratory Society (ERS) guideline^23^, we believe that the quality of the spirometry was superior. Secondly, we did not assess total expiratory time in our patients with airway obstruction. We acknowledge that vital capacity in many obstructed patients is a function of total expiratory time and variations in expiratory time may have influenced our computations. Thirdly, we did not include healthy persons or patients who have combined pulmonary fibrosis and emphysema^24^ (CPFE). Future studies, using larger numbers of patients with obstructive lung diseases, should examine the utility of the Obstructive Index for predicting the presence and severity of emphysema, and should further explore the effects of fibrosis on the concavity of the MEFV curve. Fourthly, although we used multivariate analysis to adjust for the effects of the use of two different CT scanners with different slice thicknesses, it cannot be denied that measurements of LAV might have been influenced by the differences between these scanners. However, we also did the same analysis excluding patients who were scanned using the VCT, and we confirmed that the Obstructive Index was still a significant factor associated with LAV% (Supplementary Table S6). Lastly, there may be cases of bronchiolitis obliterans syndrome^25^ with severe airway obstruction and air trapping, or endobronchial tumors^26^ affecting the Obstructive Index.

In conclusion, Obstructive Index was the strongest predictor of the extent of emphysema, independently of other spirometric measurements, in patients with obstructive lung diseases in a stable condition. Although the Obstructive Index is not a reflection of the entire information that can be deduced from the shape of the MEFV curve, it can be easily calculated from the MEFV curve and we believe that this parameter could be used for evaluating the extent of emphysema in clinical practice.

## Methods

### Ethics

This retrospective study complied with the requirements of the Declaration of Helsinki, as well as with the Ethical Guidelines for Medical and Health Research Involving Human Subjects, which was issued in Japan in December of 2014 and revised in February of 2017. The latter waives the need for obtaining informed consent for retrospective collection of anonymized data and stipulates that subjects be notified of the information required for the study. Approval was granted by the ethics committee for clinical research of Tsukuba Medical Centre Hospital (IRB 2016-054, 2017-008).

### Patients

We retrospectively screened patients who underwent both chest CT and spirometry, on separate occasions within a 3-month period, and included 133 patients who were diagnosed with COPD (N = 65), ACO (N = 15), or BA (N = 53) with varying levels of airflow obstruction (Fig. 1). All patients were evaluated and treated by board-certified respiratory physicians. At the time of examination, all patients were in a stable condition, with appropriate treatments, such as long-acting muscarinic antagonist, long-acting β agonist, and/or inhaled corticosteroids according to the treatment guidelines^27–29^. The definition of each obstructive lung disease and smoking status are shown in Supplementary Appendix 1. The patients were all Japanese and were covered by public medical insurance.

### Spirometry and definition of each index

Spirometry was performed with an automated electronic spirometer SYSTEM 21 (Minato Medical Science Co., Ltd, Osaka, Japan), with the patient in a sitting position; bronchodilators were withheld before spirometry. These tests were conducted and evaluated by well-trained technicians following the statement of the ATS/ERS guideline^23^. Patients performed 3 forced expiratory maneuvers and the best curve was selected for all parameters. FEV_1_% of predicted, FVC% of predicted, and lower limit of normal of FEV_1_/FVC were determined using Japanese spirometric reference values calculated with the lambda-mu-sigma (LMS) methods published by the Japanese Respiratory Society^30^. As the variability in an individual’s lung volume and airway size could affect the concavity of the MEFV curve^31^, we included FEF_25–75_/FVC^32^, which is an index of dysanapsis^31,33^, to evaluate the curvilinearity of the MEFV curve.

The Obstructive Index was calculated by dividing FVC by the volume-difference between two points of half of the peak expiratory flow on the MEFV curve (Fig. 6)^14^.

### Visual assessment of MEFV curve concavity

The shape of the MEFV curves was visually divided into four groups according to Jayamanne’s^12^ and Healy’s criteria^13^: AC, Int, C, and N, (Fig. 2). The AC has an abrupt decrease in flow rate and an inflection point that occurs at less than 50% of the PEF and within the first 25% of the FVC. The Int is similar to the AC but meets only 1 of the AC criteria. The C exhibits a gradual decrease in the descending limb of the MEFV curve. The visual patterns of the MEFV curves were assessed by 3 respiratory physicians reaching a consensus.

### CT scan protocols

CT examinations were performed during a breath-hold at deep inspiration. Of the patients, 103 were scanned with an Aquilion™ CT device (Canon Medical Systems Corporation, Otawara, Tochigi, Japan), and 30 were examined using a Light Speed VCT™ (GE Healthcare, Waukesha, WI). Images were obtained at a slice thickness of 2.0 mm (Aquilion) or 1.25 mm (VCT), with a scan time of 400 or 500 milliseconds (Aquilion) or 400 milliseconds (VCT), tube voltage of 120 kV, and with autoexposure. The CT images were reconstructed with a smoothing kernel (FC10 and Standard).

### CT measurements of the lung

The DICOM formatted data of each patient were used to analyze lung measurements. Using SYNAPSE VINCENT™ ver 4.6 (FUJIFILM Medical Corp., Tokyo, Japan), LAV% at −960 Hounsfield units, divided by the whole-lung volume, and WA% of the right B^1^ and the right B^8^ were measured^5–7,9,10,34^.

### Statistical analyses

All statistical analyses were performed using JMP™ version 14.1.0 for Windows (SAS Institute Inc., Cary, NC). Differences in characteristics of subjects and QCT measurements among obstructive lung diseases were analyzed using omnibus tests (Kruskal-Wallis Tests or Chi-square tests), followed by nonparametric comparisons for all pairs using the Steel-Dwass method. Spearman’s rank correlation was used to evaluate the relationship between QCT measures and spirometric indices. To investigate the factors associated with LAV%, multivariate regression analysis was performed. To determine the optimal cut-off values of the Obstructive Index for predicting the presence of emphysema, we constructed ROC curves and estimated AUC by levels of LAV%. The Youden index^35^ was used to identify the Obstructive Index cut-off values that maximized sensitivity and specificity. *P* values less than 0.05 were considered significant.

## Supplementary information

Supplementary Information

## Data Availability

The datasets analyzed during the study are available from the corresponding author upon reasonable request.
