# Supplementary material for: The Concavity of the Maximal Expiratory Flow–Volume Curve Reflects the Extent of Emphysema in Obstructive Lung Diseases
Source: Sci Rep. 2019 Sep 11;9:13159. doi: 10.1038/s41598-019-49591-2 (PMC6739348; doi:10.1038/s41598-019-49591-2)
Supplement: Supplementary file 1 — Supplementary Information [file 41598_2019_49591_MOESM1_ESM.pdf]

The Concavity of the Maximal Expiratory Flow–Volume Curve Reflects the Extent of Emphysema in Obstructive Lung Diseases

Fumi Mochizuki, MD; Hiroaki Iijima, MD, PhD; Azusa Watanabe, MD; Naoya Tanabe, MD, PhD; Susumu Sato, MD, PhD; Masanari Shiigai, MD; Keiji Fujiwara, MD; Takafumi Shimada, MD; Hiroichi Ishikawa, MD, PhD; Jun Kanazawa, MD; Yohei Yatagai, MD, PhD; Hironori Masuko, MD, PhD; Tohru Sakamoto, MD, PhD; Shigeo Muro, MD, PhD; and Nobuyuki Hizawa, MD, PhD

Appendix 1.

Definition of smoking status and smoking index

Current smokers were defined as those who currently smoke at least 1 cigarette per day or have smoked 1 cigarette per week for the past year. Former smokers were defined as those who had smoked for at least 1 year but not during the previous month. All other subjects were considered as never-smokers.

The smoking indices for current and former smokers were calculated by multiplying the smoking dose (number of cigarettes smoked per day/20) by the duration (years smoked).

Diagnosis of obstructive lung diseases

We referred to the document developed by the Science Committees of Global Initiative for Asthma (GINA) and Global Initiative for Chronic Obstructive Lung Disease (GOLD) for the diagnosis of obstructive lung diseases[1-3].

Bronchial asthma (BA)[1]: Symptoms were wheezing, shortness of breath, chest tightness, cough that increases in intensity over time, together with variable expiratory airflow limitation. Positive bronchodilator reversibility (increase in forced expiratory volume in 1 second (FEV_1) of > 12% and > 200 ml from baseline, 15 minutes after 30 μg of procaterol inhalation).

Chronic obstructive pulmonary disease (COPD)[2]: Symptoms were shortness of breath, chronic cough, and sputum production. Risk factors were host factors, tobacco, occupation, and indoor/outdoor pollution. The presence of a post-bronchodilator FEV_1/forced vital capacity (FVC) < 0.70.

Asthma−COPD overlap (ACO)[3]: Persistent airflow limitation with several features associated with asthma and several features associated with COPD, as described above.

Table S1 Relationship between visual assessment of the MEFV curve, the Obstructive Index, emphysema, and airway wall thickness in patients without ACO

	AC (n = 17)	Int (n = 6)	C (n = 62)	N (n = 33)	*P* (All Groups Omnibus Test)	*P* (AC vs Int)	*P* (AC vs C)	*P* (C vs N)
Obstructive Index	8.13 (6.70 to 11.6)	4.91 (3.92 to 6.34)	3.74 (2.82 to 4.88)	2.22 (1.70 to 2.53)	< 0.0001	0.029	< 0.0001	< 0.0001
LAV%	37.3 (23.9 to 55.3)	21.0 (17.4 to 40.1)	15.8 (3.40 to 24.4)	2.20 (0.55 to 13.5)	< 0.0001	0.543	0.0009	0.0045
Right B^1 WA%	44.0 (32.0 to 51.0)	39.5 (30.0 to 59.8)	42.0 (35.0 to 50.0)	48.0 (36.5 to 55.0)	0.499	0.998	0.998	0.432
Right B^8 WA%	44.0 (35.5 to 51.0)	39.0 (20.5 to 60.8)	43.5 (33.0 to 52.0)	42.5 (32.5 to 51.5)	0.953	0.969	1.000	0.996

Data are presented as medians and interquartile ranges. All MEFV curves were assessed by a consensus reading of 3 respiratory physicians. The AC has an abrupt decrease in flow rate and an inflection point at less than 50% of peak flow rate and within the first 25% of FVC[4]. The Int was similar to the AC but met only 1 of the AC criteria[5]. The C exhibited a

gradual decrease in the descending limb of the MEFV curve[5]. The schematic representation of the type of MEFV curves is shown in Figure 2. P values less than 0.05 were considered significant.

AC, airway collapse; C, curvilinear; Int, intermediate; MEFV, maximal expiratory flow-volume; N, normal.

Table S2 Nominal logistic regression analysis results of MEFV curves which exhibit inflection point using clinical characteristics and QCT measurements in patients without ACO

	LR χ^2	*P* value
Age (y)	0.93	0.3357
Female	0.59	0.4414
Height (cm)	2.84	0.0919
BMI (kg/m^2)	0.67	0.4141
SI, ≥ 10 pack-years	0.64	0.4242
Current smoker	2.23	0.1353
LAV%	14.28	0.0002
Right B^1 WA (%)	0.01	0.9402
Right B^8 WA (%)	0.02	0.8940

The target level of the dependent variable is the presence of an inflection point in the descending limb of MEFV curve (AC and Int *vs* C and N). *P* values less than 0.05 were considered significant.

AC, airway collapse; BMI, body mass index; C, curvilinear; Int, intermediate; LR χ^2, likelihood ratio chi-square; MEFV, maximal expiratory flow-volume; N, normal; QCT, quantitative computed tomography; SI, smoking index.

Table S3 Spearman's rank correlation coefficients between spirometric and QCT measurements in patients without ACO

	LAV%		Right B^1 WA%		Right B^8 WA%	
	ρ	*P* value	ρ	*P* value	ρ	*P* value
FEV_1, L	-0.43	<0.0001	-0.32	0.0005	-0.13	0.171
FEV_1% predicted	-0.44	<0.0001	-0.19	0.0443	-0.13	0.157
Obstructive Index	0.57	<0.0001	-0.04	0.635	-0.01	0.882
FEF_{25-75}, L/s	-0.55	<0.0001	-0.16	0.078	-0.10	0.293
FEF_{25-75}/FVC	-0.61	<0.0001	-0.02	0.844	-0.07	0.476

P values less than 0.05 were considered significant.

FEF_{25-75}, forced expiratory flow between 25 and 75%; FEV_1, forced expiratory volume in 1 second; FVC, forced vital capacity; L/s, liter/second; Right B^1, right apical segmental bronchus; Right B^8, right anterior basal segmental bronchus

Table S4 Multivariate regression analysis with LAV% as the dependent variable in patients without ACO

	R^2 = 0.636, P < 0.0001	
	standardized β	*P* value
Age, y	0.04	0.678
Female	-0.24	0.0283
Height, cm	-0.17	0.106
BMI, kg/m^2	-0.25	0.0001
SI, > 10 pack-years	0.07	0.371
Current smoker	-0.03	0.689
FEV_1% predicted	-0.08	0.416
Obstructive Index	0.43	< 0.0001
FEF_{25-75} /FVC	0.08	0.481
FEV_1/FVC < LLN	0.02	0.874
CT scanner, Aquilion	0.38	< 0.0001

The LLN of FEV_1/FVC were calculated using the LMS method[6]. *P* values < 0.05 were considered significant.

ACO, asthma–COPD overlap; BMI, body mass index; COPD, chronic obstructive pulmonary disease; FEF_{25-75}, forced expiratory flow between 25 and 75%; FEV_1, forced expiratory volume in 1 second; FVC, forced vital capacity; LLN, lower limit of normal; SI, smoking index.

Table S5 Obstructive Index-based prediction of emphysema progression by area under the receiver-operating characteristic curve analysis in patients without ACO

Definition of emphysema	AUC	95% CI	Youden Index	Obstructive Index threshold
LAV 10%	0.775	0.690 to 0.859	0.406	2.60
LAV 20%	0.792	0.711 to 0.872	0.467	3.96
LAV 30%	0.828	0.745 to 0.910	0.512	4.39
LAV 40%	0.803	0.676 to 0.931	0.567	5.74

AUC, area under the curve; CI, confidence interval; LAV, the percentage of low attenuation volume to total lung volume measured by quantitative computed tomography.

Table S6 Multivariate regression analysis with LAV% as the dependent variable and using data obtained from the Aquilion[TM] CT device

	All		COPD+ACO		COPD+BA		COPD	
	R^2 = 0.585, P < 0.0001		R^2 = 0.646, P < 0.0001		R^2 = 0.605, P < 0.0001		R^2 = 0.688, P < 0.0001	
	standardized β	*P* value	standardized β	*P* value	standardized β	*P* value	standardized β	*P* value
Age, y	0.03	0.774	0.07	0.550	0.06	0.577	0.14	0.268
Female	-0.20	0.098	-0.01	0.908	-0.23	0.066	-0.02	0.868
Height, cm	-0.12	0.336	0.07	0.579	-0.13	0.302	0.10	0.477
BMI, kg/m^2	-0.29	0.0001	-0.40	< 0.0001	-0.29	0.0002	-0.32	0.0018
SI, > 10 pack-years	0.08	0.409	-0.12	0.189	0.06	0.529	-0.21	0.0311
Current smoker	-0.06	0.409	-0.07	0.469	-0.02	0.759	-0.04	0.729
FEV_1% predicted	-0.11	0.352	-0.28	0.0337	-0.15	0.231	-0.39	0.0115
Obstructive Index	0.45	< 0.0001	0.32	0.0146	0.42	< 0.0001	0.31	0.0281

FEF_{25-75} /FVC	0.02	0.877	-0.11	0.496	0.00	0.978	-0.06	0.717
FEV_1/FVC < LLN	0.01	0.929	0.15	0.166	-0.05	0.685	0.09	0.385

The LLN of FEV_1/FVC were calculated using the LMS method[6]. *P* values < 0.05 were considered significant.

ACO, asthma–COPD overlap; BMI, body mass index; COPD, chronic obstructive pulmonary disease; FEF_{25-75}, forced expiratory flow between 25 and 75%; FEV_1, forced expiratory volume in 1 second; FVC, forced vital capacity; LLN, lower limit of normal; SI, smoking index.

References

1 *Global Initiative for Chronic Obstructive Lung Disease: Global strategy for the diagnosis, management, and prevention of chronic obstructive pulmonary disease (Updated 2015)*, http://goldcopd.org/gold-2017-global-strategy-diagnosis-management-prevention-copd/.

2 *Global Initiative for Asthma: Global strategy for asthma management and prevention Updated 2015*, https://ginasthma.org/wp-content/uploads/2016/01/GINA_Report_2015_Aug11-1.pdf

3 *Diagnosis of Diseases of Chronic Airflow Limitation: Asthma COPD and Asthma - COPD Overlap Syndrome (ACOS) based on the Global Strategy for Asthma Management and the Global Strategy for the Diagnosis, Management and Prevention of Chronic Obstructive Pulmonary Diseases,* https://goldcopd.org/asthma-copd-asthma-copd-overlap-syndrome/ (2015).

4 Jayamanne, D. S., Epstein, H. & Goldring, R. M. Flow-volume curve contour in COPD: correlation with pulmonary mechanics. *Chest* **77**, 749-757, doi: 10.1378/chest.77.6.749 (1980).

5 Healy, F., Wilson, A. F. & Fairshter, R. D. Physiologic correlates of airway collapse in chronic airflow obstruction. *Chest* **85**, 476-481, doi: 10.1378/chest.85.4.476 (1984).

6 Kubota, M. *et al.* Reference values for spirometry, including vital capacity, in Japanese adults calculated with the LMS method and compared with

previous values. *Respir. Investig.* **52**, 242-250, doi: 10.1016/j.resinv.2014.03.003 (2014).

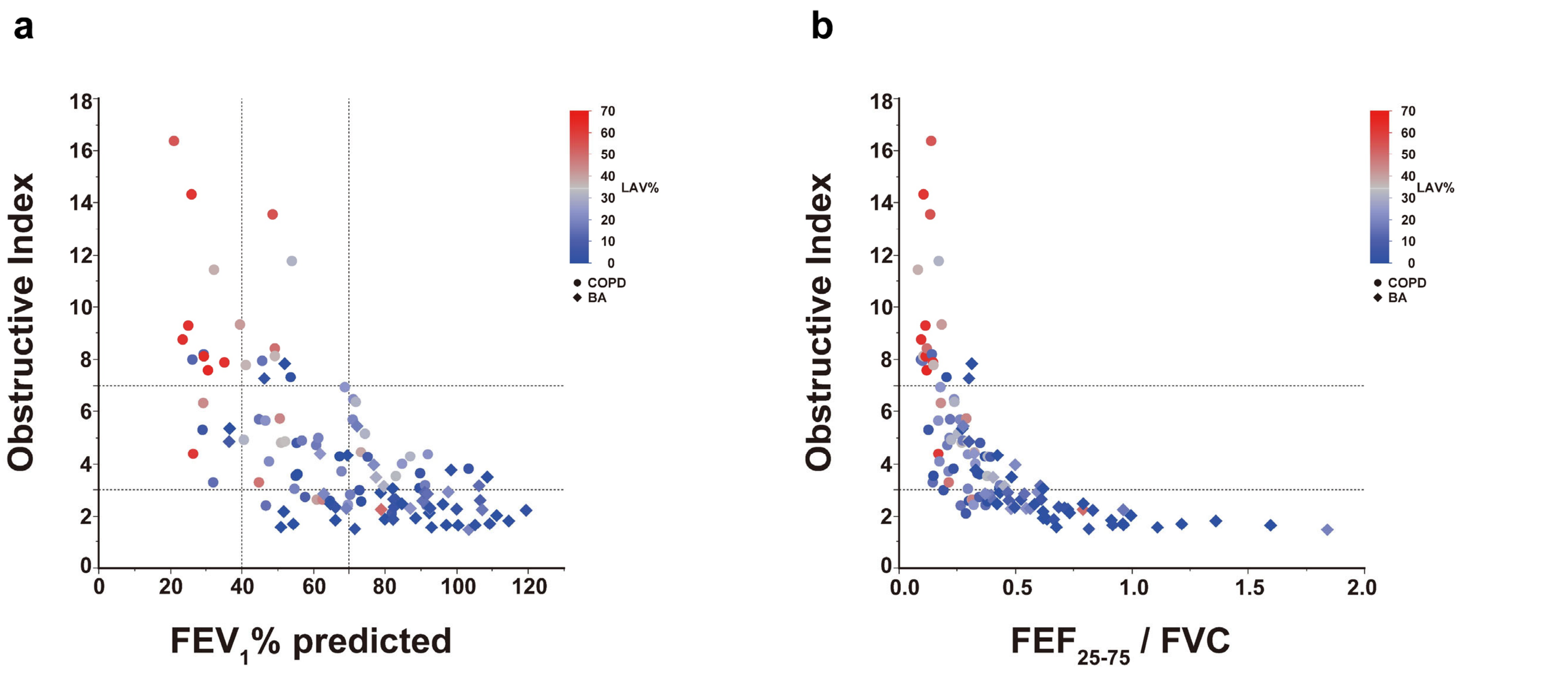

Figure S1. Correlation of the Obstructive Index with FEV_1% predicted and $FEF_{25\text{-}75}$ / FVC in patients without ACO.
a, Although a linear correlation was found between the Obstructive Index and FEV_1% predicted in patients who had high FEV_1% predicted, the linearity disappeared, and the Obstructive Index was high in COPD patients who had low FEV_1% predicted.
b, The relationship between the Obstructive Index and $FEF_{25\text{-}75}$ / FVC was hyperbolic.

ACO, asthma–COPD overlap; COPD, chronic obstructive pulmonary disease; FEV_1%, forced expiratory volume in 1 second; $FEF_{25\text{-}75}$, forced expiratory flow between 25 and 75%; FVC, forced vital capacity.

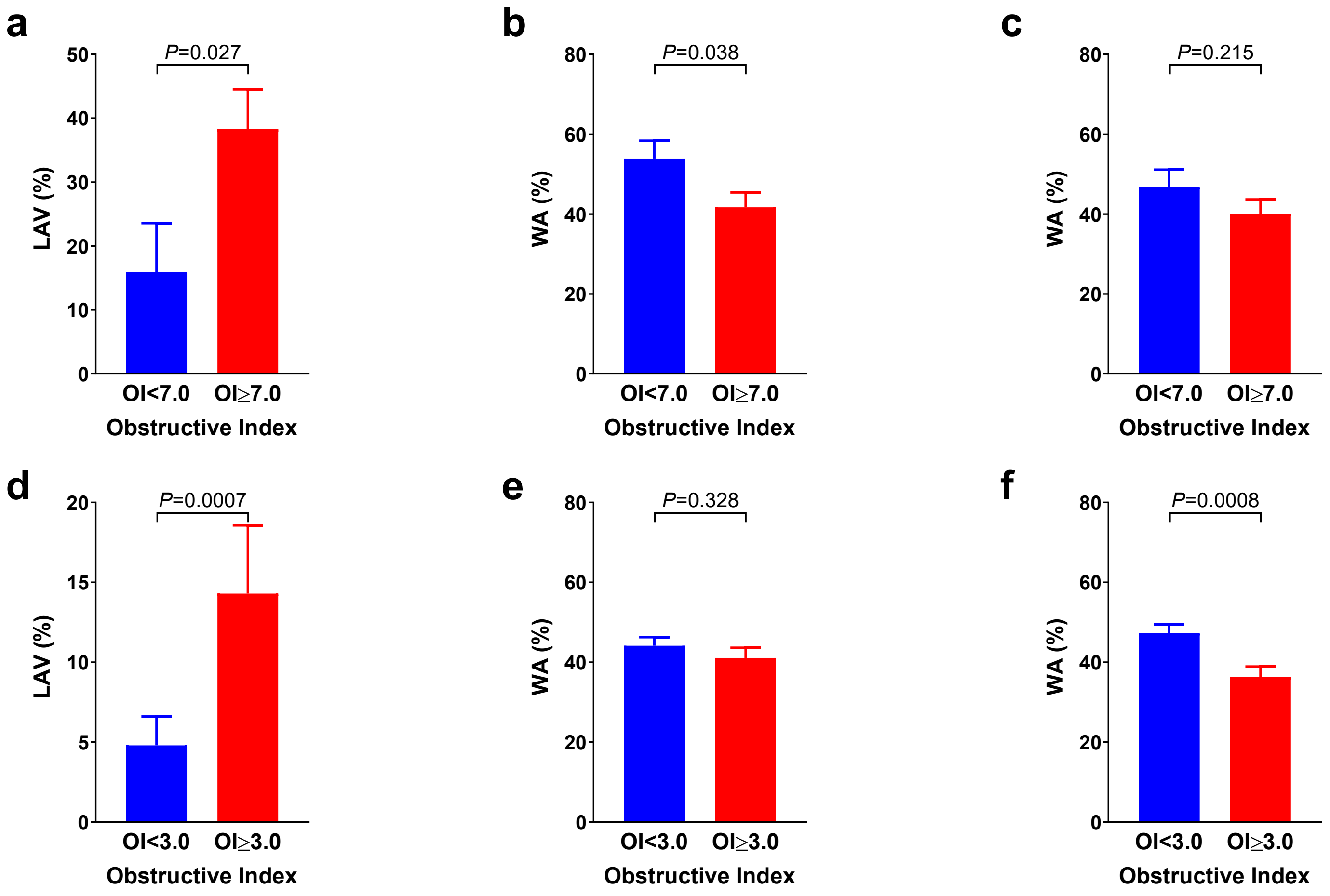

Figure S2. Comparison of QCT measurements at the cut-off value of Obstructive Index by the degree of airway obstruction in patients without ACO. Bars represent LS mean ± SEM. Values are adjusted by age and CT scanner type. There was a difference between LAV% and the Obstructive Index in patients who had either a low FEV_1% predicted (≤ 40%, a, b, c) or a high FEV_1% predicted (≥ 70%, d, e, f). The Obstructive Index <7.0, N=6 (BA = 2, COPD = 4); the Obstructive Index ≥7.0, N = 11 (BA = 0, COPD = 11); the Obstructive Index <3.0, N = 36 (BA = 31, COPD = 5), the Obstructive Index ≥3.0, N = 22 (BA = 8, COPD = 14).

ACO, asthma–COPD overlap; BA, bronchial asthma; COPD, chronic obstructive pulmonary disease; CT, computed tomography; FEV_1, forced expiratory volume in 1 second; LAV, low attenuation volume; LS, least squares; QCT, quantitative CT; SEM, standard error of the mean; WA%, the percentage of wall area.

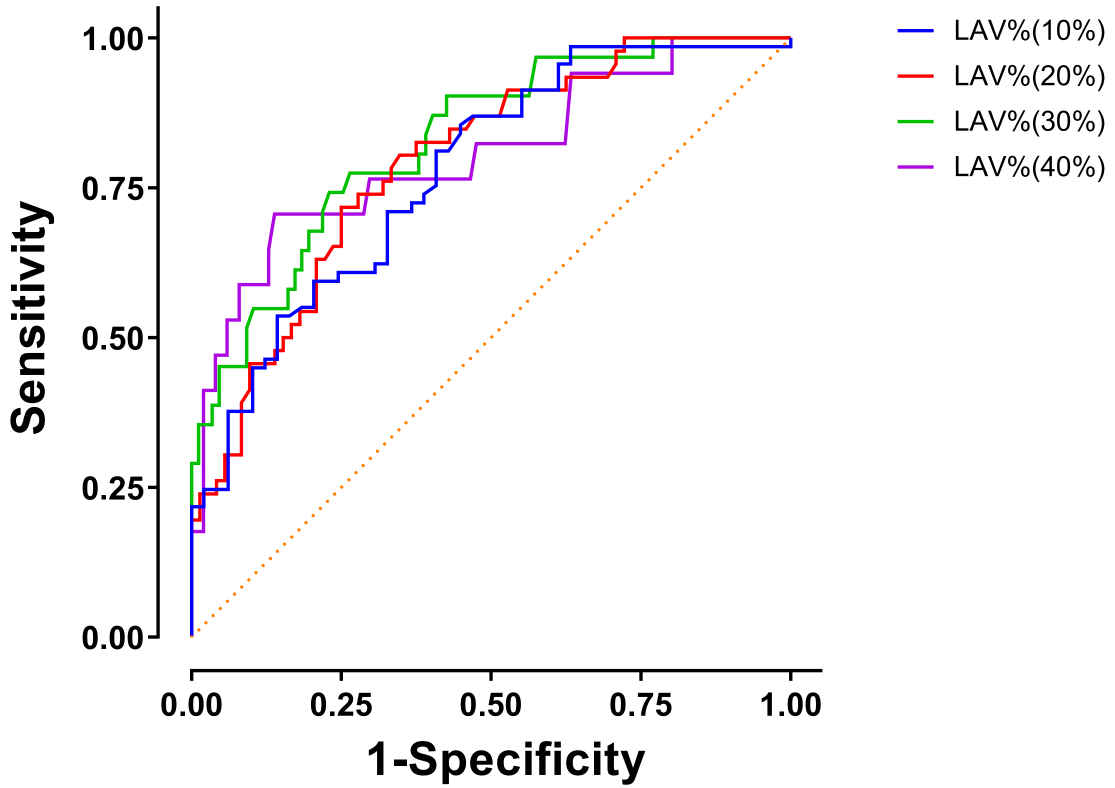

Figure S3. Comparison of LAV of total lungs for predicting the presence of emphysema by ROC analysis in patients without ACO. We evaluated emphysema based on four stages of LAV%:10%, 20%, 30%, and 40%. ROC analysis showed that a LAV of 30% and an Obstructive Index of 4.39 were the optimal cut-off values for determining the presence of emphysema in patients with COPD and BA (Table S5).

ACO, asthma–COPD overlap; BA, bronchial asthma, COPD, chronic obstructive pulmonary disease; LAV, low attenuation volume; ROC, receiver operating characteristic.
